# Homoeolog Inference Methods Requiring Bidirectional Best Hits or Synteny Miss Many Pairs

**DOI:** 10.1093/gbe/evab077

**Published:** 2021-04-19

**Authors:** Natasha Glover, Shaoline Sheppard, Christophe Dessimoz

**Affiliations:** 1 SIB Swiss Institute of Bioinformatics, Lausanne, Switzerland; 2 Center for Integrative Genomics, University of Lausanne, Switzerland; 3 Department of Computational Biology, University of Lausanne, Switzerland; 4 Global Health Institute, EPFL, Lausanne, Switzerland; 5 Department of Genetics, Evolution, and Environment, University College London, United Kingdom; 6 Department of Computer Science, University College London, United Kingdom

**Keywords:** homoeolog, *Gossypium hirsutum*, cotton, comparative genomics, best bidirectional hit, synteny

## Abstract

Homoeologs are pairs of genes or chromosomes in the same species that originated by speciation and were brought back together in the same genome by allopolyploidization. Bioinformatic methods for accurate homoeology inference are crucial for studying the evolutionary consequences of polyploidization, and homoeology is typically inferred on the basis of bidirectional best hit (BBH) and/or positional conservation (synteny). However, these methods neglect the fact that genes can duplicate and move, both prior to and after the allopolyploidization event. These duplications and movements can result in many-to-many and/or nonsyntenic homoeologs—which thus remain undetected and unstudied. Here, using the allotetraploid upland cotton (*Gossypium hirsutum*) as a case study, we show that conventional approaches indeed miss a substantial proportion of homoeologs. Additionally, we found that many of the missed pairs of homoeologs are broadly and highly expressed. A gene ontology analysis revealed a high proportion of the nonsyntenic and non-BBH homoeologs to be involved in protein translation and are likely to contribute to the functional repertoire of cotton. Thus, from an evolutionary and functional genomics standpoint, choosing a homoeolog inference method which does not solely rely on 1:1 relationship cardinality or synteny is crucial for not missing these potentially important homoeolog pairs.

**Significance**

Best bidirectional hit, with or without an added synteny criteria, is often used to infer pairs of homoeologs of an allopolyploid. However, this technique misses duplicated or nonsyntenic homoeolog pairs. We show that up to 26% of cotton homoeologs are missed in *Gossypium hirsutum* when using a combined BBH and synteny approach for inference, and considering the length and expression pattern of the genes involved, many of them appear to be functional.

## Introduction

Polyploidy is widespread in plants, with virtually all angiosperms having undergone at least one round of polyploidization in their evolutionary history (De Bodt et al. 2005; Van de Peer et al. 2009). In extant plant taxa, nearly a quarter are polyploids, half of which are estimated to be allopolyploids (Barker et al. 2016). However, there are still many unanswered questions about polyploids, specifically about how polyploidy generates evolutionary novelty, and why it may be advantageous for plants (Van de Peer et al. 2017). Using homoeologs in large-scale computational studies may provide insight into the evolutionary consequences of polyploidization.

With an abundance of whole-genome sequences, of which more and more are polyploids (Kyriakidou et al. 2018), homoeolog inference is key in many downstream analyses. Just as accurate orthology prediction is a cornerstone of comparative genomics, accurate homoeology prediction is important for comparing subgenomes of polyploids. Indeed, homoeologs can be loosely thought of as orthologs between subgenomes in an allopolyploid species, as they both arise from speciation events (Glover et al. 2016). Best bidirectional hit, also known as best reciprocal hit or bidirectional best hit, henceforth abbreviated as BBH, is a common technique for comparing subgenomes; the mutually closest sequences between two subgenomes, measured by a sequence similarity criterion such as BLAST score or E-value, are taken as the homoeologs. Homoeolog inference methods relying on BBH are widely used, for example, in *G. hirsutum* (upland cotton) (Zhang et al. 2015; Hu et al. 2019), *Triticum aestivum* (bread wheat) (International Wheat Genome Sequencing Consortium [IWGSC] 2014; Takahagi et al. 2018), and *Arachis hypogaea* (peanut) (Clevenger et al. 2016). Furthermore, a loose-to-stringent synteny requirement, or positional conservation, of homoeologs along the chromosome is frequently applied in addition to BBH (Tang et al. 2008), including studies in *Arachis hypogaea* (Bertioli et al. 2019)*, Brassica napus* (rapeseed) (Chalhoub et al. 2014), *Fistulifera solaris* (oleaginous diatom) (Tanaka et al. 2015; Nomaguchi et al. 2018), and *G. hirsutum* (Li et al. 2015).

The use of these BBH and synteny criteria is not without merit. Allopolyploidization involves a hybridization of two closely related species followed by a whole-genome doubling. Thus, homoeologs are generally close in evolutionary distance, because most diploid progenitors (and thus subgenomes) diverged a relatively short time ago. One can then assume that the majority of true homoeologs exist in a 1:1 relationship between subgenomes. The additional synteny requirement adds extra evidence to any given pair; genes which remain in their syntenic, ancestral position are also likely to be true homoeologs.

However, from a conceptual standpoint, several evolutionary processes could disrupt the 1:1 relationship cardinality or positional conservation of homoeologs. First, homoeologs which have undergone duplication would not remain at a 1:1 relationship, but may exist at a 1:many or many:many relationship (Glover et al. 2016). Small-scale gene duplication is possible after the divergence of diploid progenitors, including after the polyploidization event. Duplicated genes are broadly looked at as the raw evolutionary material for change in genomes, and may have several fates including neofunctionalization, subfunctionalization, pseudogenization, or maintenance of functions in both copies (Zhang 2003; Conant and Wolfe 2008). Often, genes belonging to large multigene families are enriched for adaptive functions to help the organism deal with stress: tolerance or resistance to heat, cold, salt, heavy metals, antibiotics, drugs, pesticides, or pathogens, among others (reviewed in Kondrashov [2012]).

Second, small-scale gene rearrangement may interrupt the synteny of homoeologs. A growing body of evidence shows that large- and small-scale genome structural rearrangements can be a result of polyploidization (Kenton et al. 1993; Song et al. 1995; Parisod et al. 2010). Nonsyntenic genes may be functional in genomes, with evidence suggesting they could also play an adaptive role, such as in response to biotic or abiotic stress (Glover et al. 2015), or root development (Tai et al. 2017; Baldauf et al. 2020). Thus, depending on the downstream application, it is important to use a homoeolog inference method which does not disregard duplicated genes, and does not rely on synteny.

Orthologous matrix (OMA) is a method and database for inferring orthologs (Roth et al. 2008; Train et al. 2017), and recently the OMA pipeline has been adapted to also infer homoeologs in allopolyploid species (Altenhoff et al. 2015; Glover et al. 2019). Briefly, the algorithm works by performing all-against-all alignments of protein sequences between all the genomes (or in this case, subgenomes). Pairs with at least 60% alignment overlap and a significant similarity score are considered as homologs. Next, mutually closest pairs within a confidence interval between subgenomes are considered as homoeologs. This step allows for inferring duplicated genes, that is, paralogs. The last step in the OMA pipeline is a verification step: to avoid misidentifying paralogs as homoeologs due to differential gene loss, this step searches for a third genome that has retained both paralogous copies. These copies can thus act as “witnesses of nonhomoeology” (Dessimoz et al. 2006). It is important to note that OMA also makes inference mistakes, and thus homoeologs inferred by OMA are not considered a gold-standard for this study. However, it has been shown in other contexts to be rather stringent: it makes relatively few wrong predictions at the expense of missing predictions (Altenhoff and Dessimoz 2009; Altenhoff et al. 2016, 2020). Thus, we used this method to obtain a more comprehensive (if still imperfect) set of homoeologs, due to 1) OMA’s ability to infer duplicated homoeologs, and 2) synteny conservation not being a requirement of the OMA algorithm.

*Gossypium hirsutum*, or upland cotton, is one of the most important crops worldwide, producing 90% of the world’s fiber (Jenkins 2003). It has an approximately 2.3 Gb allopolyploid genome (2*n*=4×=26) (Wang et al. 2019), and has recently been sequenced, assembled, and annotated several times over (Li et al. 2015; Zhang et al. 2015; Hu et al. 2019; Wang et al. 2019; Yang et al. 2019). The *Gossypium* diploid progenitors diverged an estimated 5–10 Ma, including the “A” genome diploid species, found to be derived from Africa, and the “D” genome diploid species from the Americas. These two species were likely reunited by transoceanic dispersal of the A genome ancestor to the Americas approximately 1–2 Ma, when the A genome ancestor (resembling extant species *Gossypium arboreum)* and the D genome ancestor (resembling extant species *Gossypium raimondii*) underwent a hybridization followed by a whole-genome duplication (Wendel et al. 2012).

Here, we used *G. hirsutum* as a system to find homoeolog pairs which are missed by conventional homoeolog inference methods, namely BBH, both with or without the synteny criteria. To investigate the validity of the additional homoeologous pairs uncovered by our procedure, we compared their properties with their syntenic BBH counterparts, including levels and breadth of gene expression.

## Materials and Methods

All analyses can be found in the supplementary Jupyter notebook, Supplementary Material online.

### Genomes Used

We used the allotetraploid genome *G. hirsutum* TM-1 for the comparative analyses. The assembly and annotation used for homoeolog inference in OMA and for best bidirectional hit (BBH) were: *G. hirsutum* (AD1) Genome NAU-NBI Assembly v1.1 and Annotation v1.1 (Zhang et al. 2015). This protein annotation consisted of 70,478 genes. The genome was downloaded from: https://www.cottongen.org/species/Gossypium_hirsutum/nbi-AD1_genome_v1.1 (last accessed April 22, 2021). The file downloaded for use in this analysis did not contain alternative splice variants, so we assume the canonical/longest transcript was used.

### Homoeolog Inference by OMA

OMA infers homoeologs by using the protein annotation of the genome sequence, treating the subgenomes as separate genomes, and predicting orthologs between the subgenomes. However, a necessary prerequisite is assignment of contigs to one of the two subgenomes. Three percent of the genes were on unmapped contigs with no assigned subgenome, so they were discarded for the rest of the analysis. The remaining 97% of the genes were used for homoeolog inference. Details on the OMA algorithm can be found in Glover et al. (2019) and Train et al. (2017). The June 2019 version of the OMA database version was used; it can be downloaded in HDF5 format from https://omabrowser.org/oma/archives/All.Jun2019/ (last accessed April 22, 2021). We queried the OMA HDF5 database using the python library pyoma (available on pypi).

### Homoeolog Inference by Best Bidirectional Hit

Protein coding genes from the A and D subgenomes were used as queries in BlastP (version 2.6.0) (Altschul 1997) searches against one another. The default parameters of BLAST were used, and only the max_target_seqs = 1 was returned. Gene pairs that displayed the best reciprocal BlastP hits between the two subgenomes were extracted.

The sequence alignment coverage for pairs found uniquely by BBH was taken from the BlastP result’s subject and query coverage. Whichever was the minimum was used for plotting in the sequence alignment coverage histogram in supplementary figure 2, Supplementary Material online.

In order to determine which pairs were defined as paralogs by OMA rather than homoeologs, we queried the OMA HDF5 database using pyoma to obtain inferred paralogs in *G. hirsutum*.

### Synteny Scores

Using the method described in Glover et al. (2019), synteny scores were computed for each pair of homoeologs. Briefly, we did this by taking a window of five genes downstream and five genes upstream of each homoeolog in the pair. We then calculated the proportion of genes in each window which are homoeologous to at least one gene in the window of the homoeolog on the opposite subgenome. We then binned all the homoeolog pairs into 11 categories based on their synteny score using the python pandas library (version 1.2.0) “cut” function. The first bin includes all scores in which the synteny score is 0. The rest of the bins are right-bound, starting from the category “0–.1,” which for example means that the bin includes homoeolog pairs with synteny scores higher than >0, and ≤0.1. Homoeologs with a synteny score in the first bin were considered nonsyntenic. We plotted the synteny score distribution with windows of 20, 30, and 40 genes as comparison. The code for computing synteny scores is available in the pyoma library in the module synteny.py. Using the synteny information along with the BBH status, we divided all homoeolog pairs into four categories: *BBH & syntenic*, *non-BBH & syntenic*, *BBH & nonsyntenic*, and *non-BBH & nonsyntenic*.

### Characteristics of Homoeologs: Nb. Homoeologous Pairs, Evolutionary Distance, and Protein Length

We computed five metrics for each category of homoeolog pairs. The first metric, evolutionary distance, is a pairwise metric, as it is a characteristic of the relationship between the two genes in the homoeolog pair. Evolutionary distance is measured in point accepted mutation units (PAM), and is the amount of sequence evolution which will change on an average 1% of the amino acids.

The rest of the metrics are gene-centric, with a value for each gene in the homoeolog pair. Multigene families consist of many homoeologous pairs, with the same gene potentially involved in multiple pairs. Thus, when computing statistics on the characteristics of homoeolog pairs, the same gene may be counted multiple times. For plotting and statistics for these gene-centric metrics, we took each homoeolog category and combined all the genes comprising those pairs into a list. For each category, if a gene was present twice, we removed the redundancy so that each gene was only represented once per category. For an example, see supplementary figure 1, Supplementary Material online. This gene-centric, filtered data set was used for summary statistics and to plot the number of homoeologous pairs, protein length, expression breadth, and expression level (see Expression section of Materials and Methods). To compute the number of homoeologous pairs, we simply counted the number of homoeologous relations for each gene. The homoeologous pairs and protein lengths were obtained from the June 2019 OMA database. All summary statistics were computed with pandas version 1.2.0 (Mckinney 2011) and plots drawn with seaborn version 0.11.1 (Waskom et al. 2021). SciPy version 1.6.0 (Virtanen et al. 2020) was used to perform a Kolmogorov–Smirnov test between each pair of categories of homoeologs, with the two-sided alternative hypothesis.

### Expression

RNA-seq raw paired-end reads of several organs and leaf treated tissues of *G. hirsutum* TM-1 were downloaded from the bioproject PRJNA248163 (Zhang et al. 2015). This experiment included 12 different plant tissues where the plant was not under any stress (control conditions): leaves, seed, cotyledon, stem, petals, roots, torus, stamen, pistil, calycle, ovule, fiber. Five tissues had data for different time-point samples: fiber: 5, 10, 20, 25 days postanthesis, cotyledon: 24, 48, 72, 96, 120 h, root: 24, 48, 72, 96, 120 h, ovule: 0, 1, 3, 5, 10, 20, 25, 35 days postanthesis, and seed: 0, 5, 10 h. Thus, 32 samples in total were used for the analysis (25 time-point samples plus the calycle, leaf, petal, pistil, stamen, stem, and torus expression abundance). The reads were mapped to the *G. hirsutum* genome and quantified using Kallisto-0.46.1 (Bray et al. 2016). Bootstrap sample for the quantification was set to 100, all other parameters were set to the default in Kallisto. Genes were considered expressed when their transcripts per kilobase million (TPM) value was ≥2 TPM. The five tissues with time-point samples were averaged to find the overall mean TPM for the tissue. Expression breadth for each gene was computed by counting the number of control conditions out of the 12 which had expression. The gene-centric filtered data set described above was used for summarizing the expression breadth and expression level per homoeolog category.

### Gene Ontology Enrichment

We tested for gene ontology (GO) enrichment for each of the homoeolog categories. All genes were assigned GO annotations based on those stored in the OMA database. These GO annotations come from mapping public annotations to the cotton genes in the same Orthologous Group (Altenhoff et al. 2015). The background population considered was all the genes in the gene-centric data set (each unique gene only considered once per homoeolog category). The study sets for each enrichment test were the genes in each of the four synteny/BBH-status categories, again from the gene-centric data set. Goatools (version 1.0.15) was used to perform the GO enrichment study (Klopfenstein et al. 2018), and GO annotations were propagated from parent to children terms. Fisher’s exact test was used for computing *P* values, and they were corrected using the Bonferroni method. Only those enrichments of terms with a *P* value <0.05 were retained. Enriched GO terms were used as input for Revigo, which reduces the redundancy of lists of GO terms (Supek et al. 2011). Revigo was also used to visualize the enriched GO terms and summarize the most relevant terms, based on the TreeMap function.

### Comparison of Genes in Different Versions of Annotation to Search for Fragmentation

In order to check that shorter length genes were not just artefactual fragments due to the assembly, we compared them with a newer assembly which uses long-read sequencing technology (assumed to have less fragmentation). The long-read TM-1 genome (Yang et al. 2019) was downloaded from https://www.cottongen.org/data/download/genome_tetraploid/AD1 (last accessed April 22, 2021). The predicted coding sequences from the CRI_v1 assembly were used in a BlastN against the coding sequences from NBI_Gossypium_hirsutum_v1.1.cds.fa (Zhang et al. 2015). We then checked for exact matches between the “old” annotation genes and “new” annotation genes with the following criteria: 100% identity, 100% query coverage, 100% subject coverage, and that the name of the gene in the old assembly is the same as the name of the gene in the new assembly.

## Results

We first sought to infer a more complete set of potential homoeolog pairs which included both BBH and non-BBH pairs. We used the *G. hirsutum* TM-1 genome (Zhang et al. 2015), consisting of 70,478 annotated genes. This includes 32,032 genes mapped to subgenome A, 34,402 to subgenome D, and 4,044 genes not assigned to any subgenome. Since such assignment is required for homoeologous inference, genes without subgenome assignment were not considered in the rest of this study.

### Inferring a More Comprehensive Set of Homoeologs and Comparison with BBH

OMA is a method and database for inferring homoeologs in allopolyploid species (Altenhoff et al. 2015; Glover et al. 2019). We used this method to obtain a more comprehensive set of homoeologs, due to OMA’s ability to infer duplicated homoeologs, and synteny conservation not being a requirement of the OMA algorithm. Homoeolog inference using OMA resulted in 32,426 pairs of homoeologs between the A and D subgenomes. With OMA, any given gene can have more than one pairwise homoeologous relation. We thus consider this to be a more liberal set of putative homoeologs, which we use to compare with the BBH method with and without synteny.

**Fig. 1.**
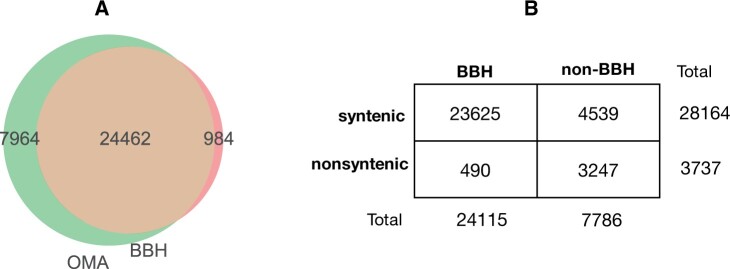
(*A*) Overlap between homoeolog pairs found with OMA (green) and pairs found with the BBH method (red). (*B*) Contingency table of the comprehensive set of homoeolog pairs from OMA, subdivided into categories based on synteny and BBH-status (*BBH & syntenic*, *BBH & nonsyntenic*, *non-BBH & syntenic*, and *non-BBH & nonsyntenic*). Only pairs for which we were able to compute a synteny score were used (31,901 out of 32,426 pairs after removing those with genes on small scaffolds). The majority of the pairs are both syntenic and BBHs, but 8,276 pairs (25.9%) are either nonsyntenic, non-BBH, or both nonsyntenic and non-BBH. Furthermore, 3,247 pairs (10.2%) are both nonsyntenic and non-BBH.

In order to compare homoeolog pairs inferred solely from BBH with the larger set of homoeologs found with OMA, we performed a BBH analysis using BlastP between the genes’ protein sequences of the A and D subgenomes in *G. hirsutum*. We found 25,446 BBHs between the A and D subgenomes. Between the 32,426 homoeolog pairs found with OMA and the 25,446 pairs found with BBH, 24,462 were identical between the two methods, which is 75.4% of the more liberal set of homoeolog pairs (OMA), and 96.1% of the BBH pairs (fig. 1*A*). Thus, the majority of the pairs detected by both methods overlaps.

There were 984 pairs found by BBH and not OMA. Upon investigation, this was determined to be for two main reasons. First, 659 (67% of the BBH-only pairs) did not pass the 60% alignment coverage threshold in the OMA algorithm to be considered as homologs (supplementary fig. 2, Supplementary Material online). Second, contrary to BBH, OMA can potentially infer “hidden paralogs,” resulting from differential gene losses. That is, if one subgenome has lost one copy of the duplicates, and the other subgenome has lost the other copy of the duplicates. In this scenario, even though the genes have originated by duplication and are truly paralogs, they are the mutually closest in sequence between subgenomes. Thus, this “witness of nonhomoeology” step in OMA searches for a third genome that retained both homoeologous copies. In this case, the genomes used to check for asymmetric gene loss were: *Theobroma cacao, Corchorus capsularis, G. arboreum*, and *G. raimondii*. Pairs that pass this test are considered to be homoeologs. Out of the remaining 325 BBH-only pairs, 206 pairs were inferred as paralogs by OMA (supplementary Jupyter notebook, Supplementary Material online). Thus, the vast majority (865; 87.9%) of the pairs found uniquely by BBH is due to either not enough overlap of genes in the alignment, or they are paralogs mistakenly inferred as homoeologs by BBH. The remaining 119 pairs are likely to be false negatives in the OMA inference.

Of particular interest for the present study, 7,964 pairs were predicted uniquely with OMA. These comprise either spurious OMA predictions, or bona fide homeologs missed by BBH. Considering the high-precision/low-recall characteristic of OMA’s algorithm in benchmarks (Altenhoff et al. 2020), our experience manually vetting OMA homoeolog calls (Glover et al. 2019), and considering that BBH can only infer at most one homoeologous counterpart per gene, our presumption is that most them are bona fide homoeologs.

### Classification of Homoeologs Based on Synteny and BBH-Status

**Fig. 2.**
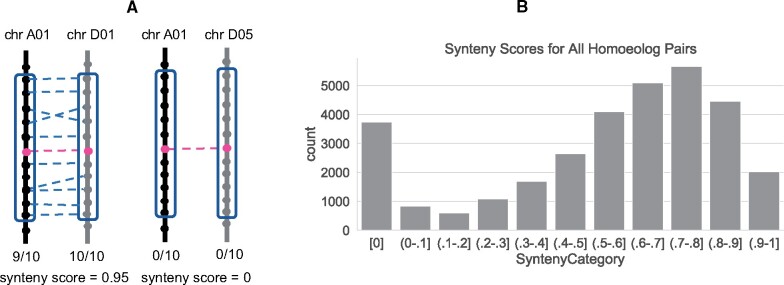
Synteny among homoeolog pairs in the cotton genome. (*A*) An example of the method for computing synteny scores. For each homoeolog pair (connected red dots), a window of ten neighboring genes around each homoeolog is formed. The synteny score is computed as the fraction of the 10 + 10 = 20 neighbors that have at least one homoeologous counterpart in the other window (blue-dotted lines). (*B*) Histogram of the synteny scores for the homoeolog pairs (*N* = 31,901). The first bin includes only synteny scores of 0. The rest of the bins include the rightmost edge.

Next, we computed a synteny score for each pair of homoeologs, using the method described in Glover et al. (2019). Briefly, we did this by taking a window of five genes downstream and five genes upstream of each homoeolog in the pair. We then computed the proportion of genes in each window which are homoeologous to at least one gene in the window of the homoeolog on the opposite subgenome (fig. 2*A*). Our criteria for computing a synteny score is that there must be at least two genes in both the windows of the homoeolog pair. Therefore, we removed 525 pairs (1.6%) which had one or both genes on a small scaffold so we could not compute synteny for them. This left 31,901 homoeolog pairs. The synteny scores for each pair ranged from 0 (completely nonsyntenic) to 1 (completely syntenic).

The distribution of the synteny scores of all homoeolog pairs found with OMA is shown in figure 2*B*. Most synteny scores have a unimodal distribution between approximately 0.5 and 1. We repeated the analysis of synteny scores on windows consisting of 20, 30, and 40 genes, but the results were largely unchanged (supplementary fig. 3, Supplementary Material online). Interestingly, there is a peak of 3,737 homoeolog pairs that had a synteny score of 0, meaning that in the windows surrounding the homoeolog pair, no other gene is homoeologous with a gene in the corresponding window on the opposite subgenome. Henceforth, we refer to these pairs with synteny scores of 0 as *nonsyntenic homoeologs* and all the rest—synteny scores >0–1—as *syntenic homoeologs*. Although most *G. hirsutum* homoeologs have a conserved synteny, 11.7% of *G. hirsutum* homoeolog pairs detected with OMA are completely nonsyntenic. These nonsyntenic pairs would have been missed if using a synteny-dependent method of homoeolog inference.

**Fig. 3.**
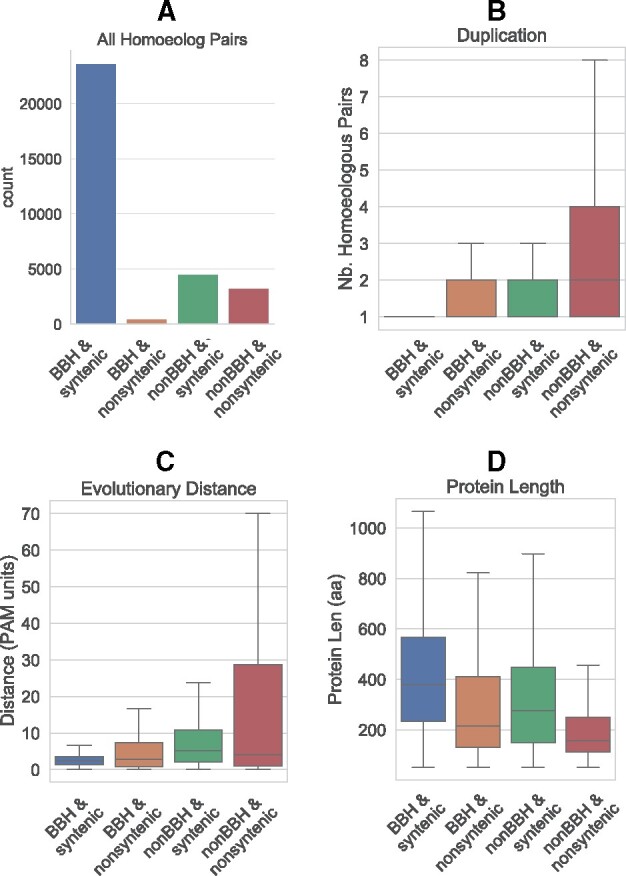
Characteristics of the four classes of homoeologs. Shown on each plot are *BBH & syntenic* (blue), *BBH & nonsyntenic* (orange), *non-BBH & syntenic* (green), and *non-BBH & nonsyntenic* (red). (*A*) Total number of pairs in each category. (*B*) Distribution of the Nb. Homoeologous Pairs, which is a proxy for the extent of duplication. A pair not having undergone duplication has a Nb. Hom. Pairs = 1. The gene-centric data set was used (see Materials and Methods). (*C*) Distribution of the evolutionary distances for all homoeolog pairs, measured in PAM units. The pair-centric dataset was used. (*D*) Distribution of protein lengths, in amino acids. The line in the middle of each boxplot represents the median, and outliers are not shown. The gene-centric dataset was used. A Kolmogorov–Smirnov test between each pair of categories in (*B–D*) showed a significant difference between all distributions (supplementary table 2, Supplementary Material online).

With both synteny and BBH information for each homoeolog pair in the OMA set (i.e., more relaxed definition of homoeology), we could then divide the set into four categories: pairs that are *BBH & syntenic*, pairs that are *BBH & nonsyntenic*, pairs that are *non-BBH & syntenic*, and pairs that are *non-BBH & nonsyntenic*. Although the majority (74%) of the pairs in the comprehensive set are both syntenic and BBHs, 8,276 pairs (26%) are either nonsyntenic, non-BBH, or both nonsyntenic and non-BBH (figs. 1*B* and 3*A*).

### Non-BBH & Nonsyntenic Homoeolog Pairs Tend to Be Duplicated, Evolutionarily Distant, and Shorter in Length

To determine whether non-BBH or nonsyntenic homoeologs are different from regular homoeologs, we next compared the characteristics of the four categories of homoeologs in terms of duplication extent, evolutionary distance, and protein length.

We used the metric “Nb. Homoeologous Pairs” to investigate if the pairs that were non-BBH are more likely to be duplicated and by how much. The Nb. Homoeologous pairs for a given gene are calculated by summing the number of homoeolog relationships it has. A Nb. Homoeologous pairs = 1 means the pair is at a 1:1 relationship between the A and D subgenomes, that is, OMA did not infer any surviving duplications for either homoeolog since the divergence of the last common ancestor. In order to not bias the results by counting the same gene multiple times per BBH category (supplementary fig. 1, Supplementary Material online), each category’s genes were filtered to take only one representative. Note that all 1:1 pairs are BBHs, but not all BBHs are 1:1. A BBH relationship between a pair of genes does not necessarily mean there are no other duplicates.

We observed that the homoeolog pairs that are also *BBH & syntenic* are far less likely to be duplicated genes (or to have undergone duplication) (fig. 3*B*). The median and mean Nb. Homoeologous Pairs for the *BBH & syntenic* category was 1 (indicating that most of the pairs were in a single copy on each subgenome, at a 1:1 relationship). The *BBH & nonsyntenic* and the *non-BBH & syntenic* categories had similar distributions, with a median of 1 and a mean of 2–2.4 Nb. Homoeologus Pairs. The last category, the *non-BBH & nonsyntenic* category had the highest median and mean (2 and 3.2, respectively) (supplementary table 1, Supplementary Material online). A Kolmogorov–Smirnov test indicated a significant difference between the distributions of every pair of homoeolog categories (*P*≤2.23e-05, supplementary table 2, Supplementary Material online). This increasing extent of duplication when considering nonsyntenic and non-BBH homoeolog pairs is shown in figure 3*B*. Thus, the non-BBH genes that would have been missed belong to larger multigene families.

Additionally, we measured evolutionary distance in PAM units, which is the amount of sequence evolution which will change on an average 1% of the amino acids. The homoeolog pairs that are *BBH & syntenic* had the lowest median and mean evolutionary distance (2.4 and 2.8 PAM units, respectively), indicating more sequence conservation and slower evolutionary rate (fig. 3*C* and supplementary table 1, Supplementary Material online). The pairs with a medium evolutionary distance were the *BBH & non-syntenic* and *non-BBH & syntenic* categories (median: 2.8–5.2, mean: 9.0–10.1 PAM units). The *non-BBH & nonsyntenic* category had a similar median (4 PAM), but the highest mean (20.8 PAM) evolutionary distance. A Kolmogorov–Smirnov test indicated a significant difference between the distributions of every pair of homoeolog categories (*P*≤3.37e-10, supplementary table 2, Supplementary Material online). This indicates that in general, non-BBH genes evolve faster than the BBH genes, and nonsyntenic genes evolve faster than the syntenic genes.

Finally, we looked at the protein length of the genes in each of the four categories. Once again, the *BBH & syntenic* category distinguished itself by having the highest median protein length compared with the rest (median 378 aa; fig. 3*D* and supplementary table 1, Supplementary Material online). The *BBH & nonsyntenic* and *non-BBH & syntenic* categories had midrange protein lengths (medians: 216–276 aa). The *non-BBH & nonsyntenic* genes had the lowest median protein length (157 aa). The same trend held true when comparing means (supplementary table 1, Supplementary Material online), and a Kolmogorov–Smirnov test indicated a significant difference between the distribution of every pair of homoeolog categories (*P*≤1.23e-10, supplementary table 2, Supplementary Material online).

At first sight, the shorter average length of non-BBH genes suggests that many of them are artefactual gene fragments due to assembly errors. To test this hypothesis, we took advantage of an improved upland cotton genome, obtained using long-read technology and recently released (CR1_v1; Yang et al. 2019). We checked whether the short genes in our study were artefactual fragments by seeing if several “separate” genes in the older assembly mapped to the same gene in the newer assembly. 99.99% of the genes in the old annotation were exact matches to genes in the new annotation (100% BlastN identity, 100% query coverage, 100% subject coverage; supplementary Jupyter notebook, Supplementary Material online). Thus, we concluded that artefactual fragmentation is not a main reason we see shorter genes.

### Genes Missed by BBH and Synteny Are Expressed

As described above, the non-BBHs and nonsyntenic genes tend to be more duplicated, have more sequence divergence, and a shorter protein length. This raises the question: Are the non-BBH and/or nonsyntenic genes functional? Using gene expression as a prima facie indicator for functionality, we used RNA-seq transcriptome data tested from 12 plant tissues (Zhang et al. 2015) to compare the expression of the different categories.

**Fig. 4.**
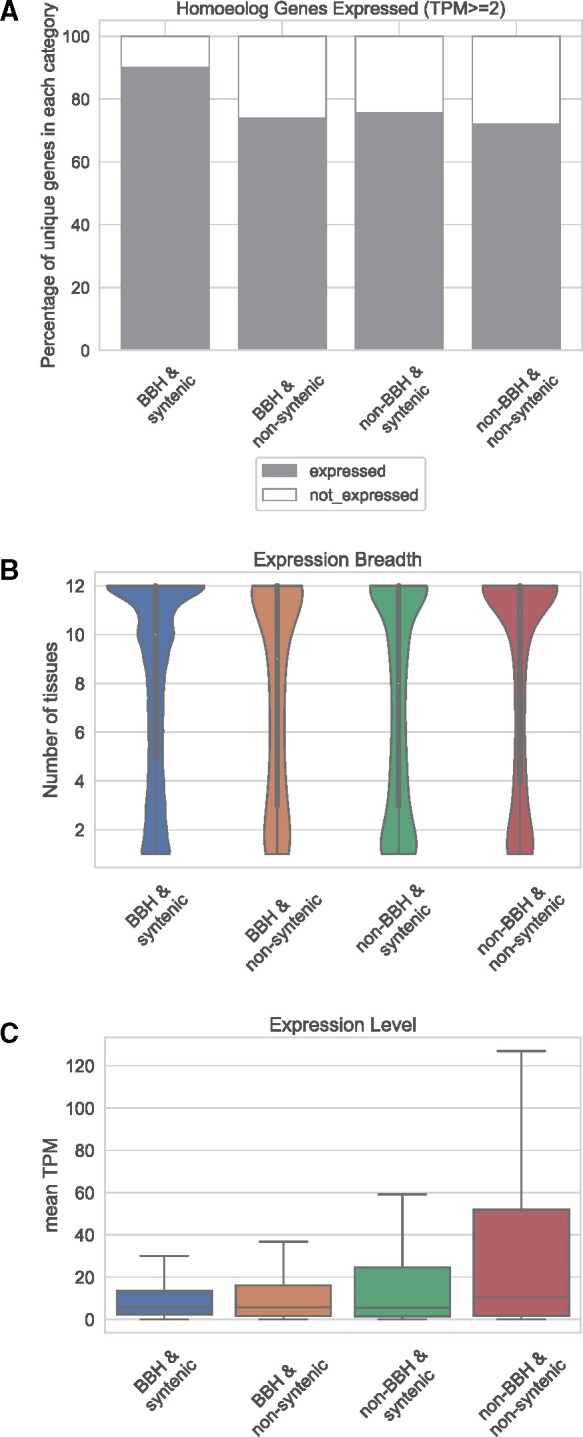
Expression analysis of the *Gossypium hirsutum* homoeolog pairs, grouped by synteny/BBH-status. Only genes with a transcripts per kilobase million value (TPM) ≥2 were considered expressed. (*A*) Survey of percentage of genes per category expressed or not. (*B*) Expression breadth, that is, number of tissues, in control conditions, in which expression was detected. The violin plot shows the density curve for each homoeolog category, where the width of the curve represents the estimated frequency of data points. (*C*) Expression level, or the mean TPM, averaged across all 12 tissues. For (*B* and *C*), only genes which were expressed at all are shown, and the filtered gene-centric data set was used. Outliers are not shown.

First, we looked at how many genes in each homoeolog category showed expression at all (TPM ≥2) (fig. 4*A*). For this, we used the gene-centric homoeolog data set, with each gene represented at most once per category. The *BBH & syntenic* category had the most genes expressed, at 89.9%. The three remaining categories (*BBH & nonsyntenic, non-BBH & syntenic*, and *non-BBH & nonsyntenic*) had similar expression patterns: between 72% and 76% of genes expressed. Thus, even though fewer of the *non-BBH & nonsyntenic* homoeolog pairs were expressed, over 70% of the genes showed some expression in at least one of the 12 tissues tested.

Next, we considered expression breadth, that is, the number of tissues out of the 12 in which the homoeologous genes are expressed. Only considering those genes that were expressed at all, the *non-BBH & syntenic* genes and the *BBH & nonsyntenic* had very similar distributions for the expression breadth (median: 8–9 tissues, mean: 7.2–7.7). A Kolmogorov–Smirnov test indicated a significant difference between these categories, but with a relatively high *P* value (*P* = 0.0331; supplementary table 2, Supplementary Material online). The *BBH & syntenic* genes had a high expression breadth, (median=10 tissues, mean 8.1). Interestingly, the *non-BBH & nonsyntenic* category had an expression breadth profile which resembled the most conserved genes (*BBH & syntenic*), with a median expression breadth of 11 and mean of 8.4 tissues (fig. 4*B* and supplementary table 1, Supplementary Material online). The distributions of all the categories were significantly different from all the rest at a *P*≤0.0331 (supplementary table 2, Supplementary Material online). The results indicate that of the nonsyntenic and non-BBH genes expressed, they are expressed relatively broadly across different tissues.

Lastly, we assessed the strength of expression in TPM. Again, we only considered unique genes once per category. All categories had similar median homoeolog expression levels (5.5–10.5 TPM) (fig. 4*C*). However, the *non-BBH & nonsyntenic* category had more than double the mean expression level of the most conserved category (*BBH & syntenic*), at 40.5 TPM compared with 16.2 TPM (supplementary table 1, Supplementary Material online). The 75% percentile and maximum (not including outliers) was much higher for the *non-BBH & nonsyntenic* compared with the others (fig. 4*C*). For expression level, a Kolmogorov–Smirnov test indicated a significant difference between all categories (*P*≤0.00402; supplementary table 2, Supplementary Material online).

### Nonsyntenic and Non-BBH Homoeologs Are Enriched for Translation Functions

With each category of homoeologs, we performed a GO enrichment to search for differences in putative biological functions between categories. In the *BBH & syntenic* category, we found an enrichment in 104 GO terms in total. The enriched biological processes, summarized by Revigo, include: Regulation of biological quality, biological process, RNA modification, biological regulation, organic substance metabolic process, among others (table 1 for summary and supplementary table 4, Supplementary Material online, for all enriched GO terms). The *BBH & nonsyntenic* category had the least amount of GO terms enriched, with 24, summarized as translation and ribosomal small subunit assembly for Biological Process. The *non-BBH & syntenic* category was enriched for 67 GO terms in total, summarized as: translation, nucleosome assembly, biosynthetic process, negative regulation of hydrolase activity, among others. Finally, the *non-BBH & nonsyntenic* category had the most GO terms enriched, 123. The main biological process enriched was ATP biosynthetic process, ribonucleoprotein complex assembly, positive regulation of translation, among others. Interestingly, ribosome or ribonucleoprotein complex were cellular components enriched in all categories except *BBH & syntenic*.

**Table 1:** GO Enrichment of Genes from Different Categories of Homoeologs

	Biological Process	Cellular Component	Molecular Function
BBH & syntenic	Total: 56. Regulation of biological quality, biological process, biological regulation, RNA modification, organic substance metabolic process, cellular process, phospholipid metabolic process, lipid metabolic process, metabolic process, methylation, response to acid chemical, response to stimulus, organelle organization, developmental process	Total: 12. Cytosol, cellular anatomical entity, cellular component, integral component of membrane, membrane, organelle	Total: 36. Protein-binding, molecular function, binding, sequence-specific DNA binding, DNA-binding transcription factor activity, catalytic activity, methyltransferase activity, hydrolase activity, phosphoric ester hydrolase activity, transferase activity, drug binding, zinc ion binding, catalytic activity acting on a protein
BBH & nonsyntenic	Total: 13. Translation, ribosomal small subunit assembly	Total: 4. Ribonucleoprotein complex	Total: 7. Structural constituent of ribosome, structural molecule activity, RNA–DNA hybrid ribonuclease activity
non-BBH & syntenic	Total: 28. Translation, nucleosome assembly, biosynthetic process, negative regulation of hydrolase activity, cell recognition, recognition of pollen	Total: 14. Ribosome, DNA packaging complex, protein-containing complex	Total: 25. Structural constituent of ribosome, structural molecule activity, protein heterodimerization activity, ADP binding, sulfotransferase activity, protein tag, protein phosphatase inhibitor activity, isoprenoid binding, chromatin DNA binding, P-P-bond-hydrolysis-driven protein transmembrane transporter activity
non-BBH & nonsyntenic	Total: 73. ATP biosynthetic process, biosynthetic process, ribonucleoprotein complex assembly, positive regulation of translation, energy coupled proton transport, energy coupled proton transport down electrochemical gradient, respiratory electron transport chain, ATP metabolic process	Total: 24. Ribosome, ribonucleoprotein complex, protein-containing complex, organelle, respirasome	Total: 26. Structural constituent of ribosome, structural molecule activity, rRNA binding, RNA–DNA hybrid ribonuclease activity, protein heterodimerization activity, catalytic activity acting on RNA, protein tag, nucleoside transmembrane transporter activity

Note.—The nonredundant, gene-centric data set was used for the enrichment. For each category of homoeologs, the study set was all the genes comprising the category, and the background set was all the genes in all categories. Enriched terms with a Bonferroni-corrected *P* value <0.05 were used to summarize the main GO terms with Revigo. The table shows the total number of GO terms enriched, and the representative terms defined by Revigo.

**Fig. 5.**
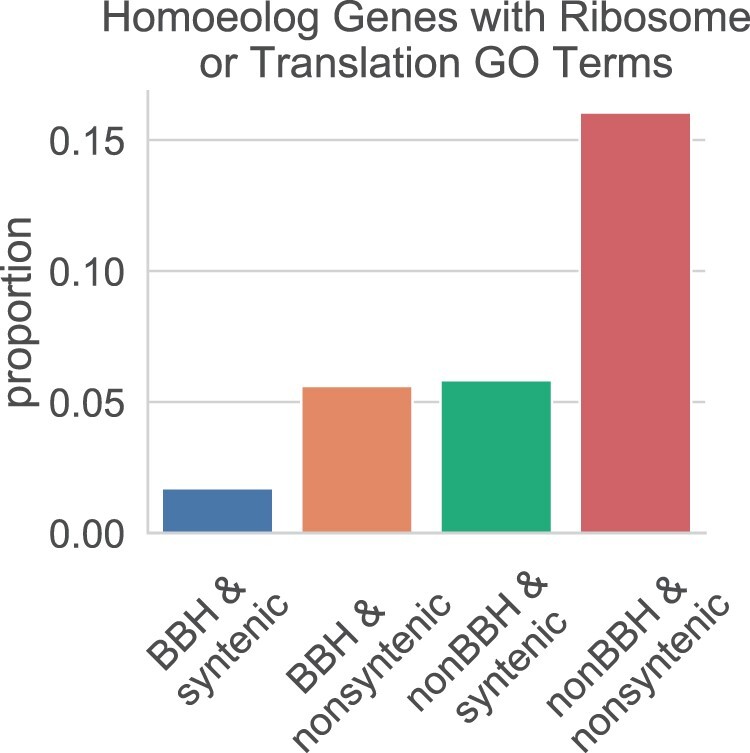
Proportion of genes per category of homoeologs which were annotated with either “translation” (GO:0006412) or “ribosome” (GO:0005840). Filtered, gene-centric data set was used.

Due to the high enrichment of processes related to translation, we summarized the proportion of the genes with GO terms “translation” and “ribosome” in each homoeolog category using the gene-centric data set. Only 2% of the homoeolog pairs in the *BBH & syntenic* category were annotated with these GO terms (fig. 5). Approximately 6% were annotated for translation or ribosome in the *non-BBH & syntenic* and *BBH & nonsyntenic* categories. However, the *non-BBH & nonsyntenic* category had a striking proportion of pairs with translation and ribosome GO terms, at 16%. All together, these results suggest that many nonsyntenic and non-BBH homoeologs could be involved in cellular translational processes.

## Discussion

In the present study, we used *G. hirsutum* to compare a commonly used method for homoeolog inference (BBH), to that with a more liberal definition (OMA). By restricting each gene to at most one homoeologous counterpart, the BBH criterion neglects the possibility that any gene duplication took place in the 5–10 Ma since the speciation of the *arboreum* (related to subgenome A) and *raimondii* (subgenome D) lineages. Although BBH generally yields few false positives, in highly duplicated genomes, it yields many false negatives. When using BBH for orthology inference, Dalquen and Dessimoz (2013) estimated 55–60% false negatives for plant and animal genomes. In this study, we show that BBH misses 26% of the homoeologs in upland cotton relative to the OMA homoeolog set.

Although we used OMA to capture the false negatives missed by BBH, any number of orthology tools could be used for this study. Like any inference method, OMA does make mistakes and is subject to trade-offs. For example, OMA works better with more and complete genomes, so more high-quality related species in the Gossypium clade could improve the inference. Additionally, we used pairwise orthologs rather than Hierarchical Orthologous Groups and that could make a difference in the number of pairs inferred (Zahn-Zabal et al. 2020). However, this would likely yield more homoeologs; that is, more group-induced pairs of homoeologs which were not inferred to have a homoeologous relation when looking at pairwise subgenome comparison alone. Furthermore, we used an older assembly based on short reads, and even though the gene annotations did not change, the synteny for some pairs might have changed by using an assembly with long reads. We attempted to mitigate this by looking only at local synteny, by comparing neighborhoods of ten genes surrounding each gene in the homoeolog pair. Even though OMA has algorithmic limitations, benchmarking studies comparing leading orthology inference tools show that OMA is rather stringent; It makes relatively few wrong predictions at the expense of missing predictions (Altenhoff et al. 2016). The consequence on this study is that by using OMA, the estimated proportion of missed homoeologs by BBH is likely to be underestimated.

By improving methodological limitations of conventional approaches, the 32,426 homoeolog pairs we found with OMA in the upland cotton TM-1 genome are considerably more than the number of pairs found in other studies: 25,358 homoeolog pairs reported in Zhang et al. (2015), 22,876 pairs reported by Li et al. (2015), and 21,419 pairs reported by Hu et al. (2019). Though there are variations in the assembly and annotation versions used across these studies, the differences are likely due to the kind of homoeologs inferred. All the methods besides OMA were based on either bidirectional best hit (BBH), with or without a synteny requirement. By contrast, OMA also inferred one-to-many or many-to-many homoeologs, which accounts for duplications which are subgenome-specific, that is, duplications which occurred after the divergence of the progenitor species. Furthermore, OMA does not have a synteny requirement with inferring homoeologs. Like BBH, synteny-based approaches have a high precision; homoeologs remaining in their ancestral position are likely to be true homoeologs. However, these positional homoeologs are just a subtype of homoeologs (Glover et al. 2016; Dewey 2011), so there will necessarily be false-negatives.

In this study, we use gene expression as a proxy for functionality, and most of the genes missed with BBH or by synteny were expressed. Although it is true that expression by itself does not necessarily indicate biological function (Kellis et al. 2014), we also considered expression breadth and expression level. Of the 72% of genes comprising *non-BBH & nonsyntenic* homoeolog pairs that were expressed, they were expressed in many conditions and at relatively high levels, suggesting that they are indeed functional in the *G. hirsutum* genome. Conversely, absence of expression does not necessarily mean no biological function. We only checked for expression in the control conditions, where the plants were not under any stress. For homoeologs where we did not find expression, it is possible that they could still be playing a role in response to certain stress conditions. Moreover, more tissues or time points may also show some levels of expression in those genes where no expression was detected in this study.

The four categories of homoeologs defined in this study could have important biological implications. The largest category of homoeologs was the *BBH & syntenic* pairs. This set of genes represents the conserved, ancestral genes. All of their characteristics indicate this: positionally conserved, the least amount of duplication and sequence divergence, and the longest protein length. They were expressed in the most number of genes and the highest number of tissues. A GO enrichment indicated that they have general, metabolic functions. This is in line with other studies, for example, in the allohexaploid *Triticum aestivum*, where homoeologs conserved at a 1:1:1 ratio were expressed at a higher level and a higher breadth than those homoeologs that had experienced duplication or loss (Juery et al. 2020).

The *BBH & nonsyntenic* and the *non-BBH & syntenic* homoeologs had similar properties for most of the metrics we looked at: a midrange evolutionary distance, protein length, number of genes expressed, expression breadth, and expression level. However, they notably differed in number; *BBH & nonsyntenic* only had 490 genes compared with the 4,539 genes in the *non-BBH & syntenic*. The *BBH & nonsyntenic* pairs are likely to be transposed genes, potentially moved by a “cut and paste” mechanism. This may be due to transposable elements, which have been shown to capture genes and move them in the genome in several species (Jiang et al. 2004; Yang and Bennetzen 2009; Catoni et al. 2019). The *BBH & nonsyntenic* genes tend to evolve faster and duplicate more than their syntenic counterparts. On the other hand, the *non-BBH & syntenic* homoeologs have stayed in their local gene neighborhood, yet are not the mutually closest hits between subgenomes, indicating duplication. These are likely to be tandem duplicates, also indicated by their relatively high Nb. homoeologous pairs.

Finally, the last category of homoeologs, the *non-BBH & nonsyntenic*, had interesting properties. These genes are duplicated *and* transposed, thus implicating a “copy and paste” mechanism. They were highly duplicated, fast-evolving, and with a smaller protein length. The difference in protein lengths between the homoeolog categories could not be explained by fragmentation caused by errors in assembly or annotation. However, the mechanism of duplication for the non-BBH categories may account for these differences. Transposable element-mediated duplication and transposition, retroduplication, and double-stranded break repair could all cause gene fragmentation (Hurles 2004; Wicker et al. 2010; Panchy et al. 2016; Catoni et al. 2019). At first glance, one might suspect the *non-BBH & nonsyntenic* homoeologs to be pseudogenes. However, over 70% of the pairs show some level of expression, and at a high level and breadth. The GO enrichment indicated a high level of translation-related functions, also indicated by over 18% having a “translation” or “ribosome” GO annotation. Thus, these represent an interesting pool of genes which warrant further investigation.

In conclusion, we find a fourth of the cotton homoeologs in the OMA data set to be non-BBH and/or nonsyntenic, making them undetectable by traditional methods and perhaps understudied. Although BBH may be appropriate for some studies, there is interesting biology of those homoeologs that are missed, and these non-BBH & nonsyntenic homoeologs do appear to be playing a role in the cotton genome. Thus, choosing a method which does not have a synteny requirement and also does not miss duplicated homoeologs is crucial to finding these pairs.

## Supplementary Material

Supplementary data are available at *Genome Biology and Evolution* online.

## Supplementary Material

evab077_Supplementary_Data
