# Supplementary material for: Homoeolog Inference Methods Requiring Bidirectional Best Hits or Synteny Miss Many Pairs
Source: Genome Biol Evol. 2021 Apr 19;13(6):evab077. doi: 10.1093/gbe/evab077 (PMC8214411; doi:10.1093/gbe/evab077)
Supplement: evab077_Supplementary_Data [file evab077_supplementary_data.zip › BBH_Supplementary_file_revised_round2_clean.docx]

# Supplemental Figure 1. Schema showing the gene-centric filtration strategy. The left-hand side shows an example of a one-to-many homoeologous relationship of the A1 gene in subgenome A to three genes in subgenomes D. Categorization of these pairs results in 1 pair that is BBH & syntenic, and 2 pairs that are non-BBH & syntenic (they are in the same local gene neighbourhood). This pairwise-centric data frame was used for computing statistics and plotting Evolutionary Distance and Synteny Score. However, we also created a gene-centric dataset by listing all the genes in each homoeolog category, then taking the set of each list. This strategy was used for computing statistics and plotting Nb. Homoeologous Pairs, Protein Length, Expression Breadth, and Expression Level.


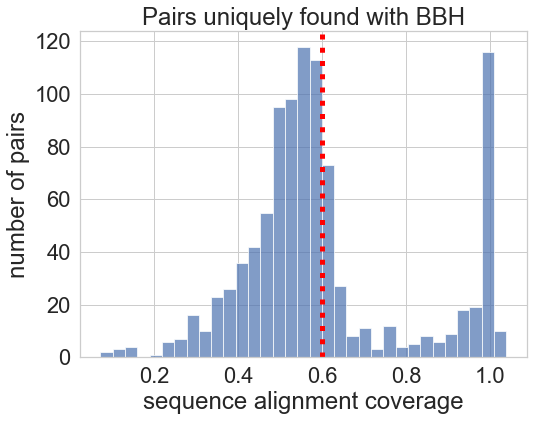


# Supplemental Figure 2. Histogram of the 984 homoeolog pairs found with BBH and not OMA. The x-axis is the sequence alignment coverage, taken from the BLASTP results. Either the query coverage or the subject coverage was used, whichever one was the minimum. The red dotted line represents a 60% alignment coverage, which is the cutoff in OMA for pairs to be considered a homolog.

**Supplemental Figure 2. Comparison of synteny scores using different window sizes.** The histogram shows the synteny scores for the homoeolog pairs for which synteny could be computed (31901). Window sizes of 20 genes (pink), 30 genes (green), and 40 genes (blue) were used around each focal pair of homoeologs between subgenomes. All bins include the rightmost edge, except the first bin, which only includes synteny scores of 0.

|  |  | BBH & syntenic | BBH & nonsyntenic | nonBBH & syntenic | nonBBH & nonsyntenic |
| --- | --- | --- | --- | --- | --- |
| Synteny Score | count | 23625 | 490 | 4539 | 3247 |
|  | mean | 0.7 | 0 | 0.53 | 0 |
|  | std | 0.19 | 0 | 0.29 | 0 |
|  | min | 0.05 | 0 | 0.05 | 0 |
|  | median | 0.7 | 0 | 0.58 | 0 |
|  | max | 1 | 0 | 1 | 0 |
| Evolutionary Distance (PAM units) | count | 23625 | 490 | 4539 | 3247 |
|  | mean | 2.81 | 10.12 | 9.03 | 20.79 |
|  | std | 2.39 | 18.71 | 11.48 | 32.68 |
|  | min | 0.05 | 0.05 | 0.05 | 0.05 |
|  | median | 2.37 | 2.76 | 5.22 | 4.01 |
|  | max | 43.06 | 142 | 104 | 223 |
| Nb. Homoeologous Pairs | count | 47250 | 980 | 4905 | 2616 |
|  | mean | 1.05 | 1.99 | 2.4 | 3.24 |
|  | std | 0.48 | 2.64 | 3.6 | 3.48 |
|  | min | 1 | 1 | 1 | 1 |
|  | median | 1 | 1 | 1 | 2 |
|  | max | 36 | 33 | 36 | 36 |
| Protein Length (aa) | count | 47250 | 980 | 4905 | 2616 |
|  | mean | 449.78 | 313.62 | 347.91 | 209.96 |
|  | std | 326.64 | 280.28 | 294.36 | 164.97 |
|  | min | 51 | 51 | 51 | 51 |
|  | median | 378 | 216 | 276 | 157 |
|  | max | 7167 | 2487 | 4349 | 1982 |
| Expression Breadth (nb. tissues) | count | 42496 | 724 | 3710 | 1884 |
|  | mean | 8.14 | 7.72 | 7.22 | 8.42 |
|  | std | 3.93 | 4.21 | 4.34 | 4.32 |
|  | min | 1 | 1 | 1 | 1 |
|  | median | 10 | 9 | 8 | 11 |
|  | max | 12 | 12 | 12 | 12 |
| Expression Level (TPM) | count | 42496 | 724 | 3710 | 1884 |
|  | mean | 16.2 | 26.43 | 29.18 | 40.53 |
|  | std | 61.1 | 76.25 | 73.96 | 72.68 |
|  | min | 0 | 0.05 | 0 | 0.04 |
|  | median | 5.91 | 5.64 | 5.52 | 10.49 |
|  | max | 5931.08 | 972.18 | 2015.71 | 934.98 |

# Supplemental Table 1. Summary statistics for the four BBH/synteny homoeolog pair categories for pairwise metrics (Synteny Score, Evolutionary Distance) and gene-centric metrics ( Protein length , Expression breadth, and Expression Level). Statistics based on genes are from the filtered dataset: for each category, all genes were counted at most once per category. For expression breadth and level statistics, only genes which had some level of expression (Transcripts Per Million >=2) were included.

#

| **Comparison** | **Metric** | **Test statistic** | **p-value** |
| --- | --- | --- | --- |
| BBH_syntenic, BBH_nonsyntenic | Evolutionary Distance | 0.23225094 | 3.00E-23 |
|  | Expression Breadth | 0.056712 | 1.97E-02 |
|  | Expression Level | 0.07925153 | 2.45E-04 |
|  | Nb. Homoeologous Pairs | 0.29095087 | 1.79E-72 |
|  | Protein Length | 0.29195767 | 5.54E-73 |
| BBH_syntenic, nonBBH_syntenic | Evolutionary Distance | 0.4139995 | 0.00E+00 |
|  | Expression Breadth | 0.11059951 | 8.32E-37 |
|  | Expression Level | 0.12144258 | 2.60E-44 |
|  | Nb. Homoeologous Pairs | 0.37421659 | 0.00E+00 |
|  | Protein Length | 0.18255114 | 4.46E-130 |
| BBH_syntenic, nonBBH_nonsyntenic | Evolutionary Distance | 0.38324749 | 0.00E+00 |
|  | Expression Breadth | 0.15844813 | 4.99E-40 |
|  | Expression Level | 0.24979211 | 1.16E-99 |
|  | Nb. Homoeologous Pairs | 0.64740721 | 0.00E+00 |
|  | Protein Length | 0.48434231 | 0.00E+00 |
| BBH_nonsyntenic, nonBBH_syntenic | Evolutionary Distance | 0.22392597 | 1.22E-15 |
|  | Expression Breadth | 0.05786809 | 3.31E-02 |
|  | Expression Level | 0.07125285 | 4.02E-03 |
|  | Nb. Homoeologous Pairs | 0.08326572 | 2.23E-05 |
|  | Protein Length | 0.11959371 | 1.23E-10 |
| BBH_nonsyntenic, nonBBH_nonsyntenic | Evolutionary Distance | 0.16174994 | 3.37E-10 |
|  | Expression Breadth | 0.1482358 | 1.70E-10 |
|  | Expression Level | 0.20120878 | 4.89E-19 |
|  | Nb. Homoeologous Pairs | 0.35645634 | 0.00E+00 |
|  | Protein Length | 0.20010454 | 0.00E+00 |
| nonBBH_syntenic, nonBBH_nonsyntenic | Evolutionary Distance | 0.20092762 | 1.82E-67 |
|  | Expression Breadth | 0.16857578 | 4.44E-16 |
|  | Expression Level | 0.13673294 | 4.44E-16 |
|  | Nb. Homoeologous Pairs | 0.27319062 | 4.33E-15 |
|  | Protein Length | 0.31529052 | 4.33E-15 |

# Supplemental Table 2. Results from pairwise Kolmogorov-Smirnov tests, which compares the distributions of the two groups of homoeolog categories. In this test, the null hypothesis is that the samples are drawn from the same distribution. A low p-value means the null hypothesis can be rejected and that the distributions are different. All p-values indicate a significant difference between groups at alpha<0.05.

# 
